# 536. Clinical Outcomes of Hospitalized COVID-19 Patients Treated with Remdesivir-NEAT ID 909REM Study

**DOI:** 10.1093/ofid/ofab466.735

**Published:** 2021-12-04

**Authors:** François Raffi, Nadir Arber, Casper Rokx, Lambert Assoumou, Pallav L Shah, Nathalie De Castro, Ameet Bakhai, Alex Soriano, Lourdes Mateu, Carlos Lumbreras, Vicente Estrada, Adrian Curran, Pierre-Olivier Sellier, Annie Duffy, Carl Fletcher, Essy Mozaffari, Richard Haubrich, Paul Hodgkins, Anton Pozniak

**Affiliations:** 1 Centre Hospitalier Universitaire de Nantes, Nantes, Pays de la Loire, France; 2 Ichilov Medical Center, Tel Aviv, Israel; 3 Erasmus University Medical Center, Rotterdam, Zuid-Holland, Netherlands; 4 Sorbonne Université, INSERM, Institut Pierre Louis d’Épidémiologie et de Santé Publique (IPLESP), Paris, Ile-de-France, France; 5 Chelsea & Westminster Hospital, National Heart & Lung Institute, Imperial College, London, UK; 6 APHP Hôpital Saint-Louis, Paris, Ile-de-France, France; 7 Royal Free London NHS Foundation Trust, London, UK; 8 Hospital Clinic de Barcelona, Barcelona, Catalonia, Spain; 9 Germans Trias I Pujol Hospital, Barcelona, Catalonia, Spain; 10 12 de Octubre University Hospital, Madrid, Spain; 11 Hospital Clinico San Carlos, Madrid, Spain; 12 Hospital Universitari Vall d’Hebron, Barcelona, Catalonia, Spain; 13 Saint-Louis/Lariboisiere Hospitals, AP-HP, Paris, Ile-de-France, France; 14 Research Organisation (KC) Ltd, London, UK; 15 Gilead Sciences, Foster City, CA; 16 Chelsea and Westminster Hospital, London, UK

## Abstract

**Background:**

There are few real-world data on the use of remdesivir (RDV) looking at timing of initiation in relation to symptom onset and severity of presenting disease.

**Methods:**

We conducted multi-country retrospective study of clinical practice and use of RDV in COVID-19 patients. De-identified medical records data were entered into an e-CRF. Primary endpoints were all-cause mortality at day 28 and hospitalization duration. We assessed time from symptom onset to RDV start and re-admission. We included adults with PCR-confirmed symptomatic COVID-19 who were hospitalized after Aug 31, 2020 and received at least 1 dose of RDV. Descriptive analyses were conducted. Kaplan-Meier methods were used to calculate the mortality rate, LogRank test to compare groups defined by severity of disease. Competing risk regression with discharge and death as competing events was used to estimate duration of hospitalization, and Gray’s test to compare the groups.

**Results:**

448 patients in 5 countries (12 sites) were included. Demographics are summarized (table) by 3 disease severity groups at baseline: no supplemental oxygen (NSO), low flow oxygen ≤6 L/min (LFO), and high-flow oxygen > 6L/min (HFO). No demographic differences were found between groups except for the higher percentage of cancer/chemotherapy patients in NSO group. Corticosteroids use was HFO 73.6%, LFO 62.7%, NSO 58.0%. Mortality rate was significantly lower in NSO, and LFO groups compared with HFO (6.2%, 10.2%, 23.6%, respectively; Fig1). Median duration of hospitalization was 9 (95%CI 8-10), 9 (8-9), 13 (10-15) days, respectively (Fig2). Median time from first symptom to RDV start was 7 days in all 3 groups. Patients started RDV on day 1 of hospitalization in HFO and LFO and day 2 on NSO groups. And received a 5 day course (median). Readmission within 28-days of discharge was < 5% and similar across all 3 groups.

Table 1. Patients baseline characteristics and primary and secondary outcomes

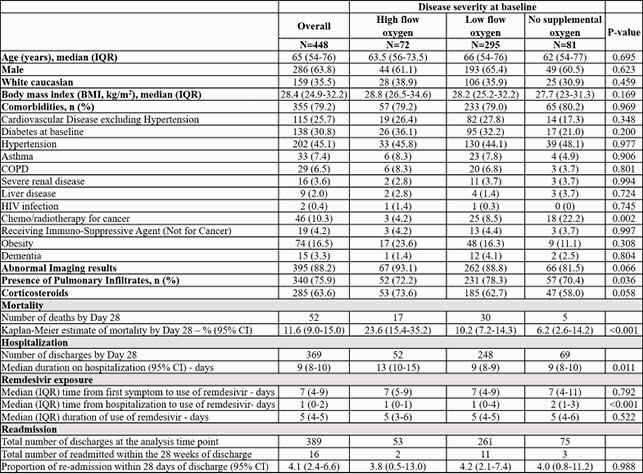

Figure 1. Kaplan-Meier estimates of mortality

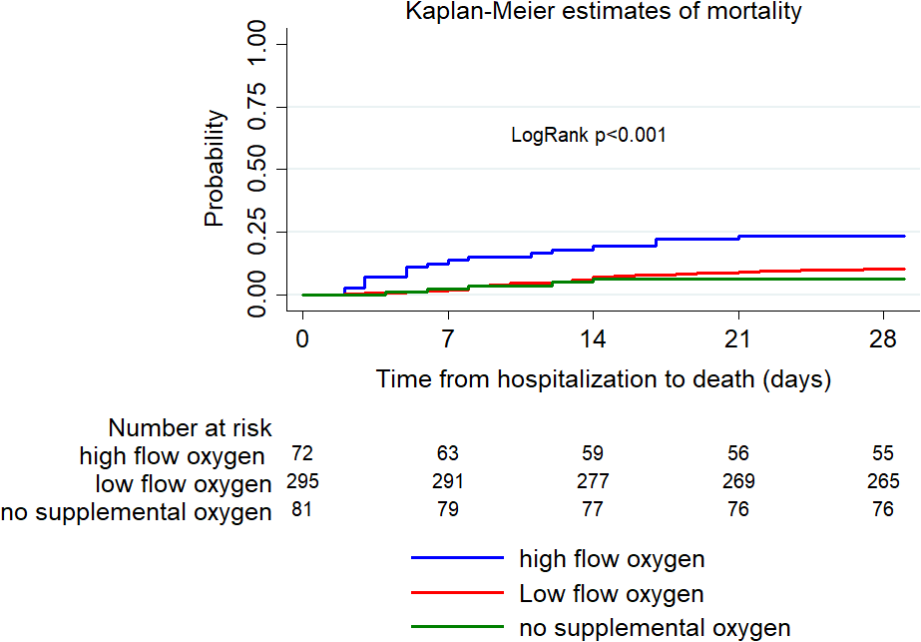

Figure 2. Competing-risks regression of discharge from hospital

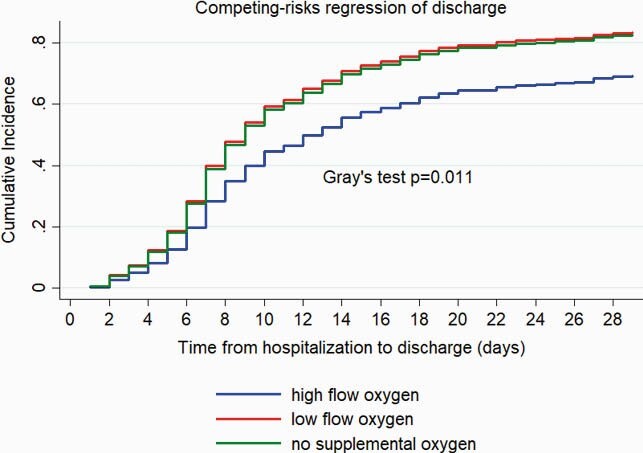

**Conclusion:**

In this real-world cohort of COVID-19 positive hospitalized patients, RDV use was consistent across countries. RDV was started within a median of 7 days from symptom within 2 days of admission and given for a median of 5 days. Higher mortality rate and duration of hospitalization was seen in the HFO group and similar rates seen in the LFO and NSO groups. Readmission was consistently low across all 3 groups.

**Disclosures:**

**François Raffi, MD, PhD**, Gilead Sciences (Consultant, Scientific Research Study Investigator, Advisor or Review Panel member)**Janssen** (Consultant)MSD (Consultant, Scientific Research Study Investigator, Advisor or Review Panel member)**Roche** (Consultant)Theratechnologies (Advisor or Review Panel member)ViiV (Consultant, Scientific Research Study Investigator, Advisor or Review Panel member) **Nadir Arber, MD, MSc, MHA**, Check cap (Consultant)**Coved cd 24** (Board Member)**Israel Innovation Authority** (Research Grant or Support)**Nucleix** (Advisor or Review Panel member)**Zion Pharmaceuticals** (Advisor or Review Panel member) **Casper Rokx, MD PhD**, Gilead Sciences (Grant/Research Support, Advisor or Review Panel member, Research Grant or Support)Merck (Grant/Research Support, Research Grant or Support)ViiV (Grant/Research Support, Advisor or Review Panel member, Research Grant or Support) **Ameet Bakhai, MBBS, MD, FRCP, FESC**, Bayer AG (Consultant, Grant/Research Support, Scientific Research Study Investigator, Advisor or Review Panel member, Research Grant or Support, Speaker's Bureau, Independent Contractor)Boehringer Ingelheim (Consultant, Grant/Research Support, Scientific Research Study Investigator, Advisor or Review Panel member, Research Grant or Support, Speaker's Bureau, Independent Contractor)Bristol-Myers Squibb (Consultant, Grant/Research Support, Scientific Research Study Investigator, Advisor or Review Panel member, Research Grant or Support, Speaker's Bureau, Independent Contractor)Daiichi-Sankyo Europe (Consultant, Grant/Research Support, Scientific Research Study Investigator, Advisor or Review Panel member, Research Grant or Support, Speaker's Bureau, Independent Contractor)Gilead Sciences (Grant/Research Support, Scientific Research Study Investigator)Janssen (Consultant, Grant/Research Support, Scientific Research Study Investigator, Advisor or Review Panel member, Research Grant or Support, Speaker's Bureau, Independent Contractor)Johnson & Johnson (Consultant, Grant/Research Support, Scientific Research Study Investigator, Advisor or Review Panel member, Research Grant or Support, Speaker's Bureau, Independent Contractor)MSD (Consultant, Grant/Research Support, Scientific Research Study Investigator, Advisor or Review Panel member, Research Grant or Support, Speaker's Bureau, Independent Contractor)Novartis (Consultant, Grant/Research Support, Scientific Research Study Investigator, Advisor or Review Panel member, Research Grant or Support, Speaker's Bureau, Independent Contractor)Pfizer (Consultant, Grant/Research Support, Scientific Research Study Investigator, Advisor or Review Panel member, Research Grant or Support, Speaker's Bureau, Independent Contractor)Roche (Consultant, Grant/Research Support, Scientific Research Study Investigator, Advisor or Review Panel member, Research Grant or Support, Speaker's Bureau, Independent Contractor)Sanofi (Consultant, Grant/Research Support, Scientific Research Study Investigator, Advisor or Review Panel member, Research Grant or Support, Speaker's Bureau, Independent Contractor) **Alex Soriano, MD**, Angelini (Speaker's Bureau)Gilead Sciences (Research Grant or Support, Speaker's Bureau)**Menarini** (Speaker's Bureau)MSD (Research Grant or Support, Speaker's Bureau)Pfizer (Research Grant or Support, Speaker's Bureau)Shionogi (Speaker's Bureau) **Carlos Lumbreras, MD, PhD**, Gilead Sciences (Grant/Research Support)MSD (Consultant) **Vicente Estrada, MD, PhD**, Gilead Sciences (Consultant, Grant/Research Support)Janssen (Advisor or Review Panel member)MSD (Consultant, Grant/Research Support)Theratechnologies (Consultant)ViiV (Consultant) **Adrian Curran, MD, PhD**, Gilead Sciences (Advisor or Review Panel member, Research Grant or Support)Janssen (Advisor or Review Panel member, Research Grant or Support)MSD (Advisor or Review Panel member, Research Grant or Support)ViiV (Advisor or Review Panel member, Research Grant or Support) **Essy Mozaffari, PharmD, MPH, MBA**, Gilead Sciences (Employee, Shareholder) **Richard Haubrich, MD**, Gilead Sciences (Employee, Shareholder) **Paul Hodgkins, PhD, MSc**, Gilead Sciences (Employee, Shareholder) **Anton Pozniak, MD, FRCP**, Gilead Sciences (Grant/Research Support, Scientific Research Study Investigator, Advisor or Review Panel member, Research Grant or Support)Janssen (Grant/Research Support, Research Grant or Support)Merck (Advisor or Review Panel member)Theratec (Grant/Research Support, Advisor or Review Panel member, Research Grant or Support)ViiV (Grant/Research Support, Scientific Research Study Investigator, Advisor or Review Panel member, Research Grant or Support)

